# COVID-19 prevalence among healthcare workers in Jakarta and neighbouring areas in Indonesia during early 2020 pandemic

**DOI:** 10.1080/07853890.2021.1975309

**Published:** 2021-11-16

**Authors:** Amin Soebandrio, Tina Kusumaningrum, Frilasita A. Yudhaputri, Sukma Oktavianthi, Dodi Safari, Safarina G. Malik, Khin Saw Aye Myint

**Affiliations:** a Eijkman Institute for Molecular Biology, Jakarta, Indonesia; b Faculty of Medicine, University of Indonesia, Jakarta, Indonesia

**Keywords:** COVID-19, healthcare workers, Indonesia, transmission

## Abstract

**Background:**

The COVID-19 disease has overwhelmed and disrupted healthcare services worldwide, particularly healthcare workers (HCW). HCW are essential workers performing any job in a healthcare setting who are potentially directly or indirectly exposed to infectious materials. Our retrospective cohort study aimed to determine the prevalence of COVID-19 infections among HCW in Jakarta and neighbouring areas during the first three months of the pandemic.

**Methods:**

Nasopharyngeal/oropharyngeal swab specimens from HCW working at private and public hospitals in Jakarta and neighbouring areas were screened for SARS-CoV-2 between March and May 2020. Data on demography, clinical symptoms, contact history, and personal protective equipment (PPE) use were collected using standardised forms.

**Results:**

Among 1201 specimens, 7.9% were confirmed positive for SARS-CoV-2 with the majority coming from medical doctors (48.4%) and nurses (44.2%). 64.2% of the positive cases reported to have contact with suspect/confirmed COVID-19 cases, including 32 (52.2%) with patient and 3 (6.6%) with co-worker. The symptomatic HCW had a significantly lower median Ct value as compared to their asymptomatic counterpart (*p* < .001). Tendency to have a higher prevalence of pneumonia was observed in the age group of 40 – 49 and ≥50 years old.

**Conclusion:**

Our findings highlighted the necessity to implement proper preventive and surveillance strategies for this high-risk population including adherence to strict PPE protocol and appropriate training.

**Key Message**
Healthcare workers (HCW), defined as those handling any job in a healthcare setting, are at the frontline of risk of infection as SARS-CoV-2 is easily transmitted through airborne droplets and direct contact with contaminated surfaces. The aim of our study is to attain a more comprehensive and accurate picture of the impact of COVID-19 on HCW during the earlier phase of the outbreak in Indonesia to develop effective strategies that protect the health and safety of this workforce. Our findings highlighted that COVID-19 infections in HCW were mostly acquired in healthcare settings, with significant consequences of pneumonia and hospitalisation occurring across all age groups.

## Introduction

An outbreak of 2019 novel coronavirus disease (COVID-19), a viral respiratory illness caused by severe acute respiratory syndrome coronavirus 2 (SARS-CoV-2) and designated by the World Health Organization (WHO) as a pandemic, was reported from countries globally [1]. As of October 2020, more than 40 million COVID-19 cases have been confirmed worldwide including 1,1 million deaths [2]. Jakarta has contributed up to 26% of COVID-19 cases nationally in Indonesia, and consequently, there was a distinct burden created on healthcare services in the city and its neighbouring areas.

Healthcare workers (HCW), defined as those handling any job in a healthcare setting, are at the frontline of risk of infection as SARS-CoV-2 is easily transmitted through airborne droplets and direct contact with contaminated surfaces [3]. HCW are at risk of infection through their occupational exposure and inadequate use of personal protective equipment (PPE). HCW could also potentially spread the infection to both patients they are handling and the community at or around their household if they become ill. At least 150,000 HCW were reported to be infected with SARS-CoV-2 worldwide and 1400 had died in the pandemic up until early May 2020 [4]. Based on the healthcare profile in 2019, Indonesia has 81,011 medical doctors and 354,508 nurses [5]. Although the information on COVID-19 cases in HCW in Indonesia was available through online media, the full extent of the COVID-19 burden among the Indonesian healthcare workforce is underreported, especially during the first months of the pandemic. The Indonesian Medical Association reported the deaths of 130 doctors and 92 nurses, and the Indonesian Dental Association the deaths of 9 dentists to the coronavirus from the beginning of the pandemic up to early October 2020 [6]. Those numbers are three times higher compared to the previous global scoping report in May 2020 that mentioned the infection of 174 Indonesian HCW and 55 deaths [4]. Healthcare workers play a crucial role and face a high risk of infection during the ongoing COVID-19 pandemic due to close contact with patients and/or potentially infectious co-workers, especially in an overstretched hospital system. During the early part of the COVID-19 outbreak in Indonesia, preventive measures had not yet been fully implemented. The inadequacy of the clinical setting and PPE might also have increased the risk of infection for HCW. The aim of our study is to attain a more comprehensive and accurate picture of the impact of COVID-19 on HCW during the earlier phase of the outbreak in Indonesia to develop effective strategies that protect the health and safety of this workforce.

## Materials and methods

### Study design and population

Our retrospective cohort study was performed based on available data for routine hospital contact tracing of HCW exposed to COVID-19 during the first few months of the pandemic. 1201 nasopharyngeal/oropharyngeal swabs from HCW working at 93 hospitals (79 private and 14 public hospitals) in Jakarta and neighbouring areas, collected between March and May 2020, were screened for SARS-CoV-2 at the Eijkman Institute for Molecular Biology (EIMB), Jakarta, a COVID-19 consortium laboratory in Indonesia. Testing was performed on samples submitted for standard diagnostic tests during the outbreak, irrespective of clinical symptoms. RNA extracted from upper respiratory specimens were subjected to SARS-CoV-2 specific real-time Reverse transcription-polymerase Chain Reaction (rRT-PCR) assays using a modified method that was originally developed by Charité Institute of Virology, Universitätsmedizin Berlin [7]. Data collected from standardized laboratory forms included symptom onset date, specimen collection date, contact history in the past 14 days, clinical manifestations, underlying health conditions, and personal protective equipment (PPE) use. In the absence of severity data in the laboratory forms, the clinical severity of COVID-19 was determined based only on chest X-ray (CXR) results, as lung ultrasound [8] or CT scan [9] with a higher diagnostic accuracy were not performed.

### Data analysis

Statistical analyses were carried out in R version 4.0.2 (https://www.r-project.org) with RStudio version 1.3.1073. Descriptive characteristics of the subjects were presented as the number of observations (percentage) for categorical variables or median (interquartile range) for continuous variables. The prevalence of reported symptoms was illustrated in a bar plot using the “graphic” package. We further classified the reported symptoms into 3 categories: respiratory symptoms (cough, shortness of breath, sore throat, runny nose), gastrointestinal (GI) symptoms (loss of appetite, diarrhoea, vomiting, abdominal pain), and other symptoms (fever, malaise, headache, shivering, dizziness, muscle pain, and joint pain). An UpSet plot showing the co-occurrence of the symptom groups in 73 symptomatic subjects was generated using the “ComplexHeatmap” package [10]. Factors associated with the number of symptoms were determined using both univariate and multivariate rank-based linear regression, implemented in the “Rfit” package [11]. The proportion of CXR findings based on age group was displayed in a bar plot and compared using Fisher’s exact test. Comparison of cycle threshold (Ct) value between groups was performed by using the Wilcoxon Mann − Whitney U test. The *p*-value of less than .050 was significant.

## Results

Among the 93 hospitals that sent HCW samples to EIMB, only 37 hospitals located in Jakarta and neighbouring areas, consisting of 32 private and 5 public hospitals, had positive test results for SARS-CoV-2. The swabs were taken from those who were symptomatic and from close contacts at the workplace. The total numbers of HCW in the study hospitals were not available. There were 7.9% (95/1201) HCW confirmed positive for SARS-CoV-2 by rRT-PCR. The positive cases consisted of various types of HCW: 46 medical doctors (48.4%), 42 nurses (44.2%), two laboratory staff (2.1%), 2 pharmacists (2.1%), 1 dentist (1.1%), 1 physiotherapist (1.1%), and 1 radiologist (1.1%). Seven cases reported their occupation as neurologist [1], pulmonologist [1], ENT specialist [1], surgeon [1], emergency room nurse [1], operating room nurse [1], and COVID-19 isolation ward nurse [1]. The median age for the positive cases was 36 years old and 62.1% were female. 76.8% (73/95) of positive cases were reported as symptomatic and 23.3% (22/95) as asymptomatic. For HCW positives with contact data reported, 61/95 (64.2%) had contact with suspected/confirmed COVID-19 cases, 32/61 (52.5%) and 3/61 (6.6%) had contact with patient and co-worker respectively. Among the HCW patients with data available on health status at the time of sample collection: 1 (1.1%) passed away, 18 (18.9%) were hospitalised (all age groups; Supplementary Figure S1), and 54 (56.8%) were not hospitalised. Severe outcomes occurred in all age groups with one death reported from a 27-year-old medical doctor with no underlying condition (Table 1). There were 8 HCW reported with one underlying conditions of either cardiovascular, diabetes mellitus, or asthma.

**Figure 1.**
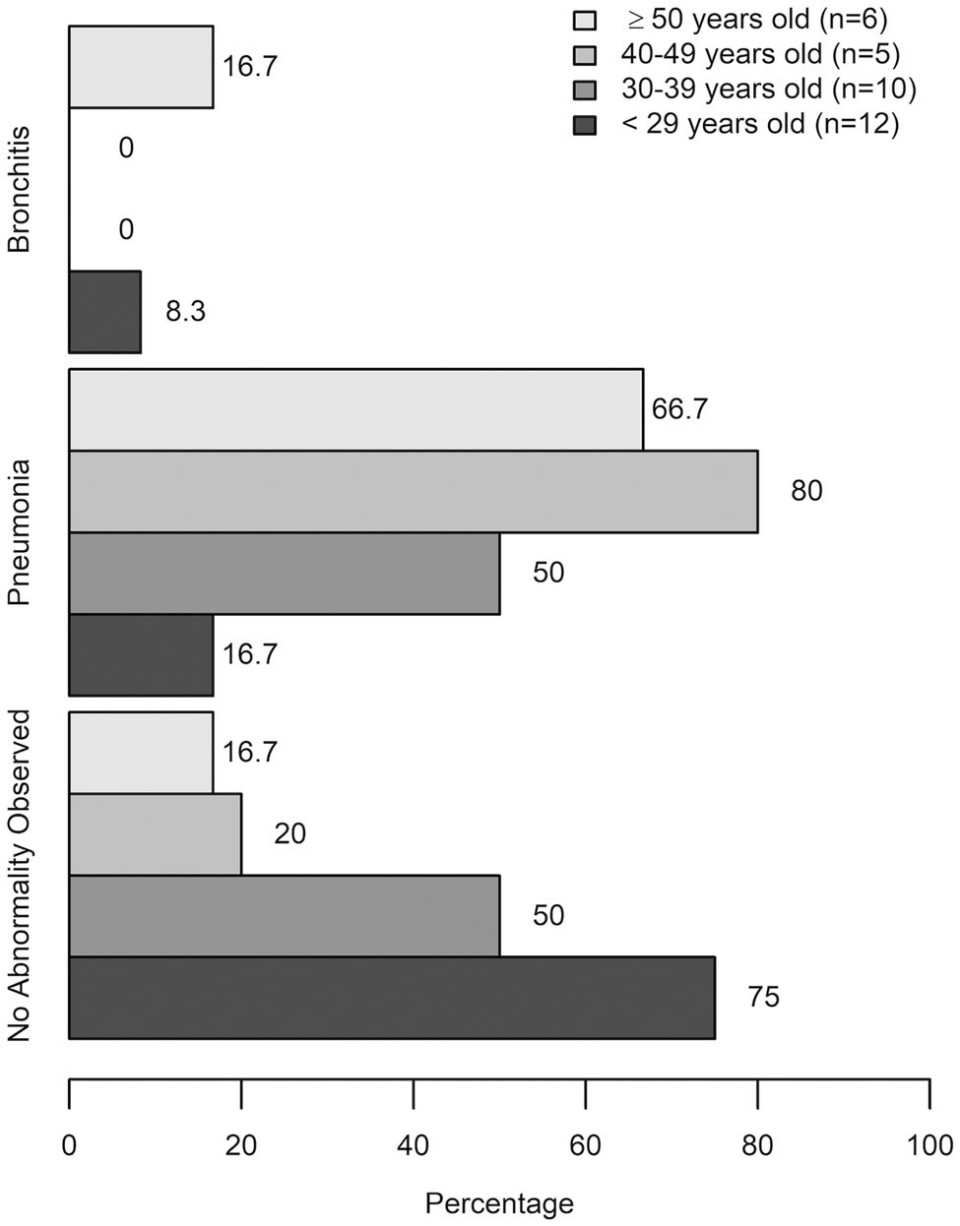
Prevalence of chest X-ray findings in the 33 HCW, stratified by age group based on median age (36 years years old). The prevalence between below and above the median age (36 years years old) groups was compared using the Fisher’s exact test.

**Table 1.** Demographic characteristics of the healthcare workers with positive SARS-Cov-2 laboratory results from March to May 2020.

Characteristic	Total (*n* = 95), *n* (%)	Asymptomatic (*n* = 22), *n* (%)	Symptomatic (*n* = 73), *n* (%)
Gender			
Male	36 (37.9)	11 (50.0)	25 (34.2)
Female	59 (62.1)	11 (50.0)	48 (65.8)
Age group			
<29 years years	30 (31.6)	8 (36.4)	22 (30.1)^a^
30–39 years years	31 (32.6)	7 (31.8)	24 (32.9)
40–49 years years	18 (18.9)	4 (18.2)	14 (19.2)
≥50 years years	16 (16.8)	3 (13.6)	13 (17.8)
Hospital location			
West Jakarta	16 (16.8)	4 (18.2)	12 (16.4)
Central Jakarta	11 (11.6)	0 (0.0)	11 (15.1)
South Jakarta	23 (24.2)	2 (9.1)	21 (28.8)
East Jakarta	2 (2.1)	1 (4.5)	1 (1.4)
North Jakarta	4 (4.2)	1 (4.5)	3 (4.1)
Bekasi	2 (2.1)	0 (0.0)	2 (2.7)
Bogor	1 (1.1)	0 (0.0)	1 (1.4)
Depok	11 (11.6)	5 (22.7)	6 (8.2)
Tangerang	20 (21.1)	7 (31.8)	13 (17.8)
South Tangerang	5 (5.3)	2 (9.1)	3 (4.1)
Type of HCW			
Dentist	1 (1.1)	0 (0.0)	1 (1.4)
Medical doctor	46 (48.4)	13 (59.1)	33 (45.2)
Laboratory analyst	2 (2.1)	1 (4.5)	1 (1.4)
Nurse	42 (44.2)	7 (31.8)	35 (47.9)
Pharmacist	2 (2.1)	1 (4.5)	1 (1.4)
Physiotherapist	1 (1.1)	0 (0.0)	1 (1.4)
Radiographer	1 (1.1)	0 (0.0)	1 (1.4)
Close contact with suspect/confirmed COVID-19			
No	13 (13.7)	3 (13.6)	10 (13.7)
Yes	61 (64.2)	14 (63.6)	47 (64.4)
Patient	32 (52.5)	9 (64.3)	23 (48.9)
Co-worker	3 (4.9)	1 (7.1)	2 (4.2)
Family	1 (1.6)	0 (0.0)	1 (2.1)
Friend	1 (1.6)	0 (0.0)	1 (2.1)
Unknown	24 (39.3)	4 (28.6)	20 (42.6)
No available data	21 (22.1)	5 (22.7)	16 (21.9)
Health status at the time of sample collection			
Not hospitalized	54 (56.8)	19 (86.4)	35 (47.9)
Hospitalized	18 (18.9)	0 (0.0)	18 (24.7)
Passed away	1 (1.1)	0 (0.0)	1 (1.4)
No available data	22 (23.2)	3 (13.6)	19 (26.0)

^a^Including 1 death.

Chest X-ray data were available from 33 out of 95 positive cases, which were reported as: no abnormality observed 16/33 (48.5%), pneumonia 15/33 (45.4%), and bronchitis 2/33 (6.1%). Further categorization based on age group showed a tendency of higher pneumonia prevalence in the 40–49 years old group (4/5, 80%) and ≥50 years old group (4/6, 66.7%), although this finding did not reach statistical significance (*p* = .065) (Figure 1).

**Figure 2.**
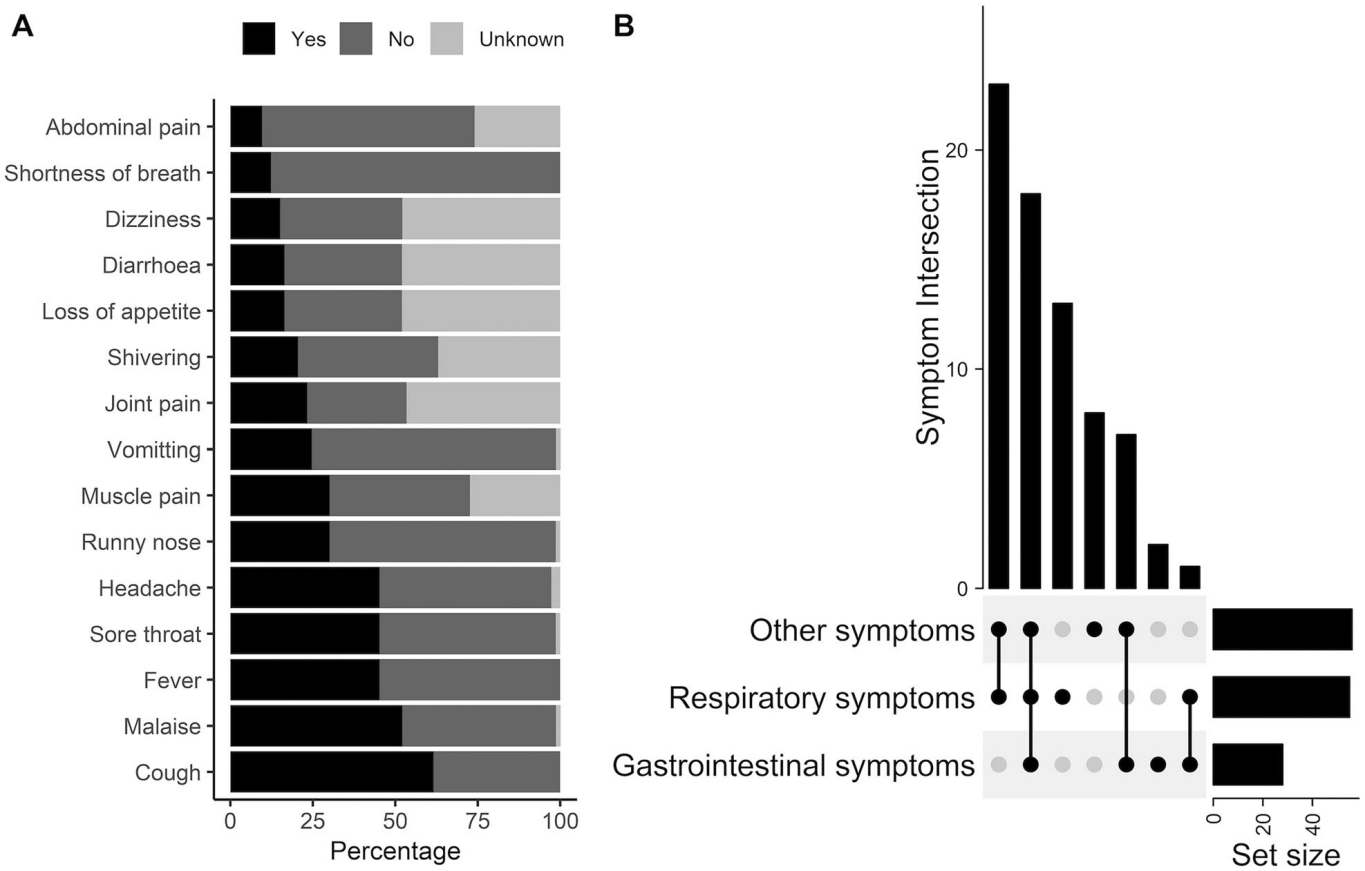
Reported COVID-19 symptoms. (A) Prevalence of reported symptoms. Bar plot showing the prevalence of symptoms for the 73 symptomatic HCW with positive SARS-CoV-2 laboratory results from March to May 2020. (B) Co-occurrence of reported symptoms in the 73 symptomatic HCW. The top bar chart shows the number of subjects who reported some combination of symptoms. Underneath is a matrix of dots represents the combination of symptoms, and the connecting lines indicate which symptoms are being combined. Classification of reported symptoms: respiratory symptoms include cough, shortness of breath, sore throat, runny nose; gastrointestinal symptoms include loss of appetite, diarrhoea, vomiting, abdominal pain; other symptoms include fever, malaise, headache, shivering, dizziness, muscle pain, join pain. The UpSet plot was generated using the “ComplexHeatmap” package.

The most relevant clinical symptoms recorded from symptomatic HCW positive cases were 45 cough (61.6%), 38 malaise (52.1%), 33 fever (45.2%), 33 sore throat (45.2%), 33 headache (45.2%), 22 runny nose (30.1%) and 22 muscle pain (30.1%) (Figure 2(A)). Our further analysis on the co-occurrence of reported COVID-19 symptoms in all symptomatic subjects showed that the most common subset of symptoms is the combination of respiratory and other symptoms (23/73; 31.5%), followed by the combination of respiratory-gastrointestinal-other symptoms (18/73; 24.6%), and respiratory symptoms only (13/73; 17.8%) (Figure 2(B)). Details of symptoms subset is shown in Supplementary Figure S2. Further analyses showed that the age group of ≥50 years was associated with 2.50 unit increase in the number of symptoms, as compared to the age group of <29 years, independent of gender, type of HCW and contact history with suspect/confirmed COVID-19 case (Table 2).

**Table 2.** Linear regression results for factors associated with the number of reported symptoms.

	Univariate model			Multivariate model		
Variable	Coef	SE	*p*	Coef	SE	*p*
Age group						
<29 years years	Reference			Reference		
30–39 years years	0.80	0.92	NS	–0.50	0.84	NS
40–49 years years	0.80	1.07	NS	–0.50	1.01	NS
≥50 years years	2.00	1.11	NS	2.50	1.07	**.023**
Gender						
Female	Reference			Reference		
Male	1.00	0.84	NS	0.50	1.07	NS
Type of HCW						
Physicians	Reference			Reference		
Nurses	<0.01	0.70	NS	0.50	0.727	NS
Others	–2.00	1.75	NS	–1.50	1.76	NS
Close contact with suspect/confirmed COVID-19						
No	Reference			Reference		
Yes	<–0.01	1.05	NS	0.50	0.91	NS

Abbreviations: Coef: coefficient of regression; HCW: healthcare worker; NS: not significant; SE: standard error. Grouping of HCW: Physicians (dentist and medical doctors), Nurses, Others (laboratory analyst, pharmacist, physiotherapist, radiographer). Linear regression analyses were performed using rank-based linear model [9]. Multivariate model was adjusted for all variables. The significant *p*-value is in bold (*p* < .050).

**Figure 3.**
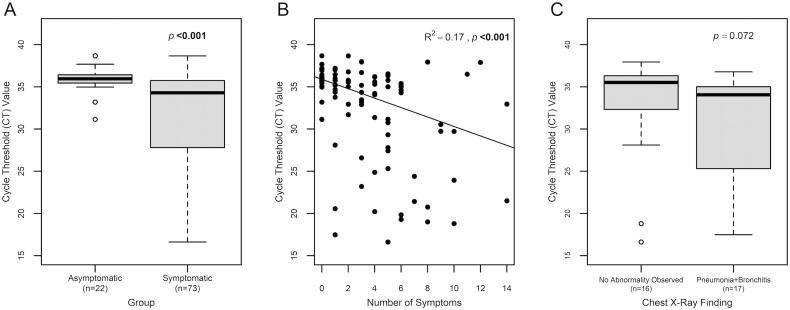
Cycle threshold (Ct) value in HCW samples. (A) Cycle threshold (Ct) value comparison of asymptomatic vs. symptomatic HCW. (B) Correlation between Ct value and number of symptoms. (C) Comparison of Ct value based on chest x-ray (CXR) findings (no abnormality observed vs. pneumonia + bronchitis). Comparison between asymptomatic and symptomatic HCW was performed using the Wilcoxon–Mann Whitney U test. Correlation analysis of Ct value and number of symptoms was performed using univariate rank-based linear regression. Comparison between CXR groups (no abnormality observed vs. pneumonia + bronchitis) was performed using the Wilcoxon–Mann Whitney U test. The significant *p*-value is in bold (*p* < .005).

The symptomatic HCW had a significantly lower median Ct value (median = 34.3, IQR = 27.8-35.8) as compared to their asymptomatic counterpart (median = 36.0, IQR = 35.5–36.4) (Wilcoxon–Mann Whitney U test: *p* < .001) (Figure 3(A)). Lower Ct value correlated with an increased number of symptoms (Rank-based linear regression: adjusted *R*^2^ = 0.17, *p* < .001) (Figure 3(B)). Further analysis on the association between Ct value and CXR findings showed that HCW with abnormal CXR (either pneumonia or bronchitis) tend to have a lower Ct value (median = 34.1, IQR = 25.3–35.0) than those with no CXR abnormality (median = 35.5, IQR = 32.9–36.3), although the relationship was not statistically significant (Wilcoxon–Mann Whitney U test: *p* = .072) (Figure 3(C)).

Data of PPE use at the hospital was available from only 26 (30.52%) positive cases with different combinations of gowns, gloves, goggles and/or masks. From the available PPE use data, 6 HCW were reported to perform aerosol-generating activities with three of them reported not using the N95 mask (Supplementary Table S1). One of 6 HCW who performed aerosol-generating activities was hospitalized with pneumonia (16.7%) (Table S2).

## Discussion

A recent study reported COVID-19 infection of 150,000 HCW globally by early May, most likely underestimated due to a lack of official data in most countries [4,12]. Currently, data on infection rates of HCW, which is critical to improving infection control and prevention measures, are limited – particularly from the Southeast Asian region, where most data is only available online [13]. In our study, 95 COVID-19 laboratories confirmed HCW were from 37 hospitals in Jakarta and neighbouring areas, mostly (24.2%) from South Jakarta City. The majority of HCW patients were females (62.1%), probably reflecting the female dominance in the Indonesian HCW workforce [5]. The clinical spectrum of COVID-19 is reported to vary from asymptomatic or mild symptomatic infections to severe respiratory symptoms and death, with older age groups generally presenting with more severe disease and higher death rates [14,15]. This is in line with our study as the older group (≥50 years old) had more varied symptoms and showed a tendency of higher prevalence of lung involvement. Our results on Ct values revealed significantly lower viral load in the asymptomatic group than in the symptomatic group, which is similar to a previously reported study [16]. This study showed that 31.5% of the positive cases was reported to experience the combination of respiratory and other symptoms. Furthermore, gastrointestinal symptoms were reported by one third of the positive cases. Previous studies reported that prevalence of gastrointestinal symptoms varied from as low as 3.8% up to 31.9% [17,18]. The data on anosmia and ageusia, common symptoms of COVID-19, as well as anxiety and depression (mental health symptoms) seen with HCW [19], were not available as they were not specified in the questionnaire designed during the early pandemic. In this study nearly 60% of HCW with COVID-19 were not hospitalised, most likely due to non-severe disease. 23% were reported as asymptomatic by the time their samples were collected with a risk of transmission to patients, co-workers, and the community if public health and social measures (e.g. quarantine procedures, social distancing, hand hygiene, mask wearing, and respiratory etiquette) were not applied. A modelling study suggested that asymptomatic cases were predicted to contribute to more than half of the COVID-19 disease transmission [20].

Although nurses were reported to be mainly affected in the published articles as they spend more time at the bedside and have more patient contact [21], the majority of the infected individuals in our study were medical doctors (48.4%), followed by nurses (44.2%). Notable in this earlier study in Jakarta was a single fatal outcome of a young medical doctor without any reported comorbidity; the data on COVID-19 related deaths in the patient population was not available. Besides the hospital staff as the front line in dealing with COVID-19 patients, laboratory personnel handling patient samples are also at risk of infection if the biosafety measures and laboratory procedures are not implemented properly [22,23].

The rate of infection of COVID-19 in HCW in Indonesia in this preliminary study (7.9%) is different from those reported in the region during the early phase of the pandemic: 5.15% from Malaysia, 19.65% from the Philippines, 3.8% from Thailand [13], 1.1% from China and those from European countries: 4.1–8.9% from the Netherlands, 9.6% from Italy, and 18% from UK [24–28]. The difference could be attributed to different types of hospitals surveyed; assays used as well as PPE usage. PPE is a critical component in reducing the transmission of COVID-19 to and from HCW when used properly and is of great importance to HCW who are at high risk of exposure to COVID-19. The safety measures adopted by the hospitals submitting specimens during the early outbreak were not known despite the national guidelines for COVID-19 prevention and control issued at that time [29]. To ensure maximal protection of this essential workforce, it is critical to address fundamental issues in providing HCW with adequate prevention and protection measures such as vigorous contract tracing, appropriate training, and sufficient supplies of PPE meeting minimum standards, including gowns, N95 respirators or facemasks, eye protection, long-sleeved gowns, and gloves for COVID-19 patient care [30]. In addition, a dedicated area to treat COVID-19 cases combined with strict infection prevention and control (IPC) implementation is considered an effective method to prevent SARS-CoV-2 transmission to HCWs [31]. A study from Hong Kong reported that a multipronged infection control strategy resulted in zero COVID −19 nosocomial infection [32]. Post-vaccination infection rates of COVID-19 among HCW in Indonesia are limited including from our study sites; however, a recent publication reported that HCW vaccinated in February 2021 with CoronaVac vaccine in one teaching hospital in Indonesia were still at risk for contracting SARS-CoV-2 infection [33].

The role of HCW in COVID-19 management is very important, therefore, it is essential to guarantee their safety. HCW should be constantly monitored for fever and respiratory symptoms for rapid identification of staff with a potential role in hospital transmission, and routinely screened for SARS-CoV-2 to prevent nosocomial viral transmission as the potential of asymptomatic carriers of SARS-CoV-2 to transmit infection was similar to that of symptomatic patients [34].

There are several notable limitations in our study: The study was a retrospective assessment of data performed on specimens submitted for a routine hospital contact tracing of COVID-19 and there were several missing data including symptoms, underlying conditions, details of occupation, health outcomes, and PPE use. Our retrospective study could not determine risk factors including the nature of contact with COVID-19 patients, behaviours associated with the development of COVID-19, adherence to recommended PPE for medical staff especially during high-risk procedures as well as identification of the source of infection. In addition, all exposed HCW were tested for SARS-CoV-2 one time only, the positive rate might have been higher if multiple swabs had been submitted. Lastly, our results from Jakarta and adjacent hospitals were mostly from private health facilities and could not be generalized to the entire country especially since only three designated COVID-19 referral hospitals in Jakarta were included in this study.

## Conclusion

To the best of our knowledge, our study provides the first comprehensive picture of COVID-19 infection of HCWs in Jakarta and neighbouring areas during the early stage of the COVID-19 outbreak. Our findings highlighted that COVID-19 infections in HCW were mostly acquired in healthcare settings, with significant consequences of pneumonia and hospitalization occurring across all age groups. However, for a better understanding of SARS-CoV-2 hospital-acquired transmission especially with the evolving pandemic, well-planned prospective studies should be conducted. Since HCW are at increased risk for infection mostly at a healthcare facility, specific requirements for their protection including strict protocols for PPE usage, early identification of infected workers, and psychological support are advisable to ensure the functioning of the basic healthcare system.

## Supplementary Material

Supplemental Material

## Data Availability

The authors confirm that the data supporting the findings of this study are available within the article [and/or] its supplementary materials.
