# Supplementary material for: COVID-19 prevalence among healthcare workers in Jakarta and neighbouring areas in Indonesia during early 2020 pandemic
Source: Ann Med. 2021 Nov 16;53(1):1896–904. doi: 10.1080/07853890.2021.1975309 (PMC8604529; doi:10.1080/07853890.2021.1975309)
Supplement: Supplemental Material [file IANN_A_1975309_SM4153.zip › Supplemental files/Supplementary Figure S1_COVID19 HCW_200521.docx]

Figure S1


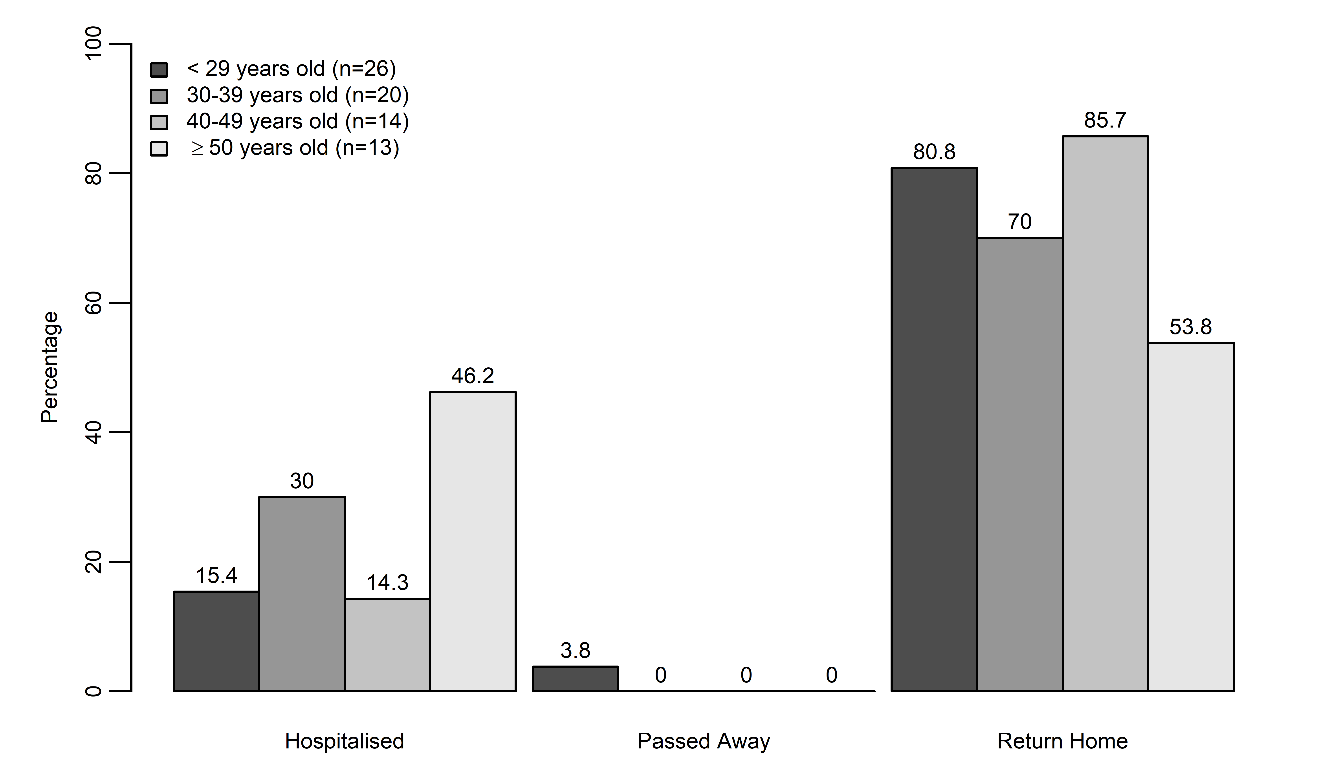


Figure S1. Distribution of health status at the time of sample collection, stratified by age group.
