# Supplementary material for: COVID-19 prevalence among healthcare workers in Jakarta and neighbouring areas in Indonesia during early 2020 pandemic
Source: Ann Med. 2021 Nov 16;53(1):1896–904. doi: 10.1080/07853890.2021.1975309 (PMC8604529; doi:10.1080/07853890.2021.1975309)
Supplement: Supplemental Material [file IANN_A_1975309_SM4153.zip › Supplemental files/Supplementary Figure S2_COVID19 HCW_200521.docx]

Figure S2


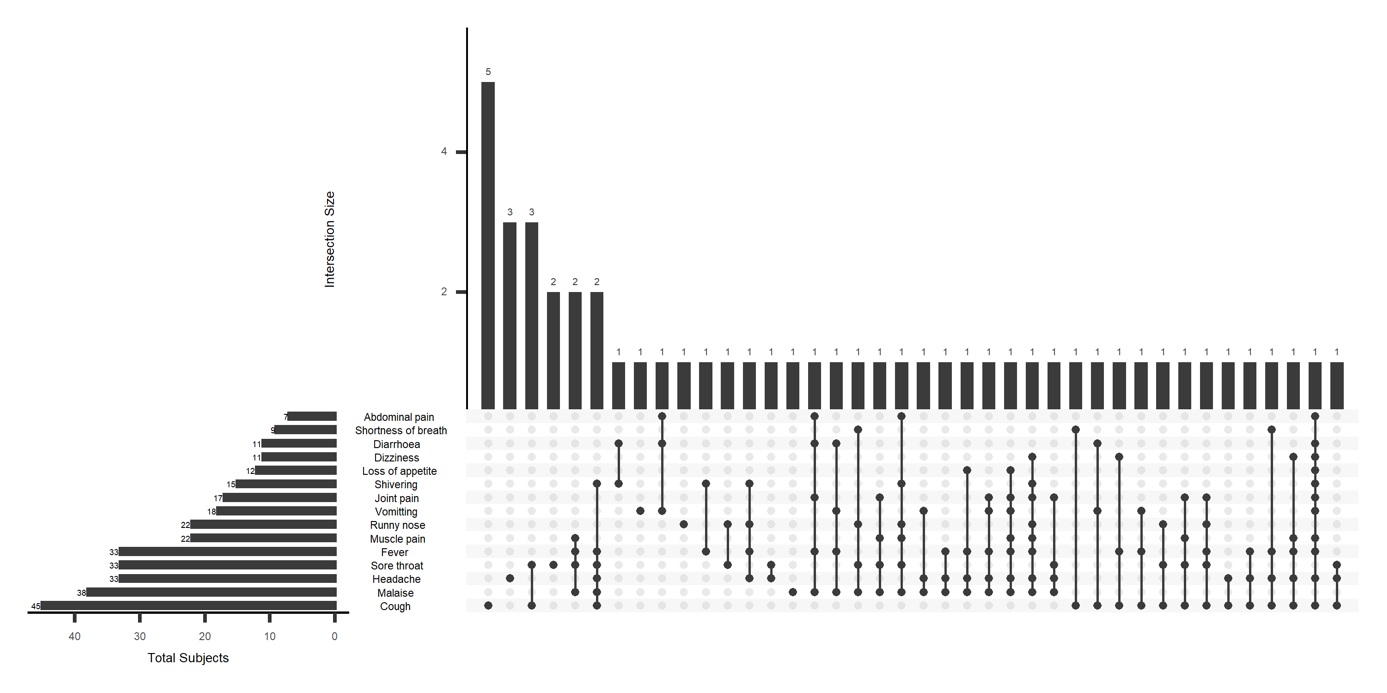


Figure S2. Co-occurrence of reported COVID-19 symptoms in the 73 symptomatic subjects. The bar chart shows the number of subjects who reported some particular combination of symptoms. Each bar is a different combination. Underneath is a graphical table showing what those combinations are. The black dots and lines show the combination of symptoms that make up each cluster or subset of symptoms. The UpSet plot was generated using the “ComplexHeatmap” package.
