# Supplementary material for: COVID-19 prevalence among healthcare workers in Jakarta and neighbouring areas in Indonesia during early 2020 pandemic
Source: Ann Med. 2021 Nov 16;53(1):1896–904. doi: 10.1080/07853890.2021.1975309 (PMC8604529; doi:10.1080/07853890.2021.1975309)
Supplement: Supplemental Material [file IANN_A_1975309_SM4153.zip › Supplemental files/Supplementary Table S1_COVID19 HCW_200521.docx]

Table S1. Reported PPE use from HCW with COVID-19 positive cases

| HCW Role | Gown  (n=13) | Surgical mask (n=24) | N95 Mask (n=11) | Gloves  (n=21) | Googles (n=15) | Performed aerosol generation activities  (n=6) |
| --- | --- | --- | --- | --- | --- | --- |
| Medical doctor (n=4) | 3 | 4 | 3 | 3 | 3 | 2 |
| Nurse (n=19) | 9 | 17 | 7 | 15 | 9 | 3* |
| Laboratory analyst (n=1) | 1 | 1 | 0 | 1 | 1 | 1 |
| Pharmacist (n=1) | 0 | 1 | 0 | 1 | 1 | 0 |
| Physiotherapist (n=1) | 0 | 1 | 1 | 1 | 1 | 0 |

*2 out of 3 nurses reported no use of N 95
