# Supplementary material for: COVID-19 prevalence among healthcare workers in Jakarta and neighbouring areas in Indonesia during early 2020 pandemic
Source: Ann Med. 2021 Nov 16;53(1):1896–904. doi: 10.1080/07853890.2021.1975309 (PMC8604529; doi:10.1080/07853890.2021.1975309)
Supplement: Supplemental Material [file IANN_A_1975309_SM4153.zip › Supplemental files/Supplementary Table S2_COVID19 HCW_200521.docx]

Table S2. Characteristic of HCW with COVID-19 positive cases, based on their involvement in aerosol generation activities

| Variable | Total (n=26) | Performed aerosol generation activities | | |
| --- | --- | --- | --- | --- |
|  |  | No (n=15) | Yes (n=6) | No available data (n=5) |
| PPE |  |  |  |  |
| Surgical mask | 24 (92.3) | 14 (93.3) | 6 (100.0) | 4 (80.0) |
| N95 mask | 11 (42.3) | 7 (46.7) | 3 (50.0) | 1 (20.0) |
| Gown | 13 (50.0) | 6 (40.0) | 4 (66.7) | 3 (60.0) |
| Gloves | 21 (80.8) | 12 (80.0) | 6 (100.0) | 3 (60.0) |
| Googles | 15 (57.7) | 9 (60.0) | 4 (66.7) | 2 (40.0) |
|  |  |  |  |  |
| Health outcome |  |  |  |  |
| Not hospitalised | 23 (88.5) | 14 (93.3) | 5 (83.3) | 4 (80.0) |
| Hospitalised | 3 (11.5) | 1 (6.7) | 1 (16.7) | 1 (20.0) |
|  |  |  |  |  |
| Symptomatic |  |  |  |  |
| No | 7 (26.9) | 4 (26.7) | 3 (50.0) | 0 (0.0) |
| Yes | 19 (73.1) | 11 (73.3) | 3 (50.0) | 5 (100.0) |
|  |  |  |  |  |
| Chest X-ray findings |  |  |  |  |
| No abnormality observed | 5 (19.2) | 4 (26.7) | 1 (16.7) | 0 (0.0) |
| Pneumonia | 5 (19.2) | 3 (20.0) | 1 (16.7) | 1 (20.0) |
| No available data | 16 (61.5) | 8 (53.3) | 4 (66.7) | 4 (80.0) |
